# Medications associated with dizziness or hypotension and adverse outcomes: an electronic health record study in older adults with dementia

**DOI:** 10.1093/ageing/afaf154

**Published:** 2025-06-08

**Authors:** Harsharon Kaur Sondh, Delia Bishara, Gayan Perera, Hitesh Shetty, Robert Stewart, Christoph Mueller

**Affiliations:** Institute of Psychiatry, Psychology and Neuroscience, King's College London, 16 De Crespigny Park, London SE5 8AB, UK; Institute of Psychiatry, Psychology and Neuroscience, King's College London, 16 De Crespigny Park, London SE5 8AB, UK; South London and Maudsley NHS Foundation Trust, London SE5 8AZ, UK; Institute of Psychiatry, Psychology and Neuroscience, King's College London, 16 De Crespigny Park, London SE5 8AB, UK; South London and Maudsley NHS Foundation Trust, London SE5 8AZ, UK; Institute of Psychiatry, Psychology and Neuroscience, King's College London, 16 De Crespigny Park, London SE5 8AB, UK; South London and Maudsley NHS Foundation Trust, London SE5 8AZ, UK; Institute of Psychiatry, Psychology and Neuroscience, King's College London, 16 De Crespigny Park, London SE5 8AB, UK; South London and Maudsley NHS Foundation Trust, London SE5 8AZ, UK

**Keywords:** dementia, dizziness, hypotension, medication, side effects, older people

## Abstract

**Background:**

Comorbidities and polypharmacy are common in people with dementia, leading to a higher risk of adverse outcomes. While the impact of anticholinergic properties has been extensively investigated, less is known about other cross-category properties of medications.

**Objective:**

To investigate whether medications with dizziness or hypotension as a side effect are associated with adverse outcomes in older adults with dementia.

**Design:**

Retrospective cohort study.

**Setting and participants:**

From a South London catchment, 15 210 patients diagnosed with dementia between 2008 and 2017.

**Methods:**

Medications with dizziness and/or hypotension listed as a side effect were compiled and quantified in the cohort. Multivariable Cox regression models were run to determine the risk of mortality, all-cause emergency hospitalisation and hospitalisation due to falls. Generalised estimating equations were applied to investigate cognitive decline. The final model adjusted for 19 potential confounders, including physical and mental health measures.

**Results:**

Of the patients, 82.2% were receiving at least one dizziness-associated medication and 71.2% at least one hypotension-associated medication. For each additional medication associated with dizziness or hypotension, there was a 4% increased risk of all-cause emergency hospitalisation. No associations were found with hospitalised falls specifically or with mortality or cognitive decline.

**Conclusion:**

Medications that potentially cause dizziness or hypotension were associated with an increased risk of hospitalisation, although not specifically hospitalisation caused by falls. More systematic attention should be paid to coprescribing around the time of dementia diagnosis and the potential for rationalising this to minimise adverse drug events.

## Key Points

- Approximately 80% of patients were taking medication causing dizziness and 70% were taking medication causing hypotension.
- There was a dose-dependent relationship between number of medications and an increased risk of emergency hospitalisation.
- There was no association between the number of medications and risk of hospitalised falls, mortality or cognitive decline.

## Introduction

Older adults with dementia frequently have co-occurring health conditions [1, 2] accompanied by an increase in the number of prescribed medications [3]. Polypharmacy increases the risk of adverse drug reactions (ADRs) [4]. ADRs are common in people with dementia [5] and result in unplanned hospitalisation, increased morbidity and mortality [6]. They have been linked to financial pressures and admissions in England projected to result in an annual cost of £2.21 billion [6].

Dizziness is a commonly reported side effect of many medications received by people with dementia, including psychotropic agents such as antidepressants and antipsychotics and nonpsychotropics such as diuretics and treatments for Parkinson’s disease [7]. Medication-associated dizziness can contribute to an increased risk of adverse outcomes [7] and is associated with other co-occurring risk factors such as a decrease in physical activity, heart disease, lower general health and polypharmacy [8]. The experience of dizziness is an independent predictor of future falls in older adults [9].

When considering their role as risk and/or outcome factors in dementia, medications are most often classified and investigated according to their primary indication—e.g. psychotropics as a group or antidepressants or antipsychotics more specifically. However, this form of categorisation, does not consider the cross-category properties of medications. For example, selective serotonin reuptake inhibitors are primarily categorised as antidepressants but have been effective in the treatment of posttraumatic stress disorder and migraines [10]. An alternative approach for understanding the effect of medication is the drug-centred model; this focuses on the modification of normal brain processing and encompasses both beneficial and adverse effects [11].

Research into cross-category ADRs in dementia has included the measurement and investigation of the anticholinergic burden; e.g. finding no association with hospitalisation and a reduced mortality risk within antidepressant and antipsychotic categories, potentially reflecting selective prescribing [11], but a higher risk of mortality associated with a high central anticholinergic burden within drugs used for urinary incontinence [12]. As well as the anticholinergic burden, recent work has also considered the use of medication with sedative properties in dementia, reporting an association with hospitalisation and accelerated cognitive decline. However, there are other ADRs such as dizziness and hypotension which have an assumed association with adverse outcomes that has not yet been fully investigated [13].

Research into polypharmacy in older adults, including dementia outcome investigations, is dominated by studies grouping medications by their primary indication. Little work has been done on the long-term outcomes of medications grouped by risk of ADRs such as dizziness and hypotension, despite the obvious clinical implications when a patient is receiving multiple agents for different indications but with overlapping ADR profiles. Better understanding of these long-term outcomes can assist clinicians in assessing and modifying risk. Hence, the aim of this study was to investigate adverse outcomes associated with the use of medications with dizziness or hypotension as a side effect in older adults with dementia.

## Methods

### Data source and sample

A retrospective cohort study was assembled using data from the South London and Maudsley NHS Foundation Trust (SLaM) Clinical Record Interactive Search (CRIS) resource. SLaM provides mental healthcare, including dementia assessment services, to a geographic catchment area of four South London boroughs (Croydon, Lambeth, Lewisham and Southwark) and ~1.3 million residents. CRIS was set up in 2008 to enable research use of deidentified data from SLaM’s electronic health records [14] and has received ethical approval as an anonymised data resource (Oxford Research Ethics Committee C, reference 23/SC/0257) [13]. It has been linked to a range of external data sources, including national hospitalisations (Hospital Episode Statistics via NHS England) and mortality data (Office for National Statistics). Data for research use are obtained from source structured fields directly and from source free text (e.g. case note entries and clinical correspondence) through the large-scale development and application of natural language processing (NLP) [14].

The extracted cohort comprised patients aged 50 or over with a first dementia diagnosis from SLaM services between 1 January 2008 and 31 December 2017. The date of dementia diagnosis served as the baseline ‘index date’ for cohort characterisation and follow-up.

### Measurements

Recorded medications are routinely assembled for CRIS from structured fields and via NLP [14]. Parallel work has taken place at SLaM to develop a means of ascertaining and quantifying medications with recognised ADRs in order to assist with clinical evaluation and intervention in polypharmacy and prescribing for vulnerable populations. This underpinned the development of the Medichec site and app [15]—first developed for central anticholinergic properties [16], subsequently supplemented with drowsiness/sedation and dizziness/hypotension and more recently extended with four other ADRs. The same medication categorisation was deployed within CRIS and the extracted cohort was characterised by the recorded use of medications with dizziness or hypotension properties as a side effect, compiled from information in the British National Formulary [17]. Recorded use of medications was ascertained in the cohort from 6 months prior to 6 months after the index dementia diagnosis date as a proxy for prevalent prescribing at dementia diagnosis. The number of recorded medications with dizziness as a side effect was modelled as a continuous variable and further categorised into an ordinal scale (0, 1, 2, 3–4, 5–6, 7+) representing the primary exposure of interest. The same grouping was used for medication causing hypotension. We had initially planned to examine associations by frequency of dizziness or hypotension according to the BNF, but of patients on at least one medication causing dizziness the vast majority (98.5%) were on a medication with dizziness as a common side effect. Similarly, of patients on at least one medication causing hypotension 73.5% were on at least one medication with hypotension as a common side effect.

Considering outcomes, date of death was identified from linked mortality records and emergency hospitalisations were ascertained from linked Hospital Episode Statistics data. From these hospitalisations, those potentially due to falls were more specifically ascertained through discharge diagnosis codes (ICD-10 (10th revision of the International Classification of Diseases) codes W00–19).

Cognitive function over the follow-up was ascertained from routinely recorded Mini Mental State Examination (MMSE) scores [18] extracted from source structured fields and via NLP. Cognitive decline rates were quantified using generalised estimating equation models [19] and applied to scores from 6 months before dementia diagnosis (index date) to 18 months after, categorised into 3-month time periods. For patients with more than one recorded MMSE score during a given 3-month period, an average score was obtained.

Covariates were obtained at the time of diagnosis or closest to that time. The sociodemographic variables extracted comprised age, gender, ethnicity (categorised into White and non-White), marital status (cohabiting vs. noncohabiting) and neighbourhood-level Index of Multiple Deprivation [20]. The MMSE score closest to the diagnosis date was also extracted. Data from routinely completed Health of the Nation Outcome Scales (HoNOS) [21] were extracted as measures of mental health symptoms and functioning. The HoNOS is a standard measure used routinely in UK mental health and dementia services and each constituent subscale is rated from 0 (no problem) to 4 (severe problems). For this study, scale scores were dichotomised to ‘no or minor problem’ (scores 0 and 1) and ‘mild to severe problem’ (scores 2 to 4), apart from the physical illness/disability subscale, which was grouped into 0–1, 2 and 3–4, as a measure of comorbidity. The presence of any hospitalisation with circulatory diseases (ICD-10 diagnoses: I20–25, I50, I60–69) in the 2 years before dementia diagnosis was also collected. Recorded acetylcholinesterase inhibitor (AChEI) use within the first 6 months after index data was ascertained due to AChEIs’ associations with cognitive trajectories [22], survival [23] and hospitalisations due to vascular events [24]. The anticholinergic effect on the cognition scale [25] was applied to all medications recorded within 6 months either side of the index diagnosis as a measure of the anticholinergic burden.

### Statistical analyses

STATA, version 18, software (StataCorp LP) was used for all the analyses. Patients were followed up until mortality, first emergency hospitalisation, first fall-related hospitalisation or 31 March 2018, as a censoring point. Associations with mortality, first hospitalisation and hospitalised fall were evaluated using the following three Cox regression models: (i) an unadjusted model (crude), (ii) a model adjusted for age, gender, ethnicity, marital status, MMSE score at diagnosis, deprivation score and central anticholinergic burden and (iii) a model adjusted for all variables in model 2 plus HoNOS symptoms and functioning scores, AChEI receipt and prior cardiovascular hospitalisation. We initially modelled the number of medications associated with dizziness or hypotension as a continuous variable. In a second step, we compared five exposure groups (1, 2, 3–4, 5–6, 7+ medications causing dizziness or hypotension) to a control group of patients not receiving medications causing dizziness or hypotension. MMSE score trajectories were only examined applying the latter approach using a generalised estimating equation and the confounder adjustments described above. Supplementary analyses were carried out with other causes of hospitalisation using the seven most common primary discharge diagnoses according to ICD-10 chapters (R, J, N, S, I, K, and M).

Of patients included in the Cox regression models 32% had missing data for at least one of the covariates. Missingness was assumed to be at random, and missing values for ethnicity, marital status, MMSE score closest to dementia diagnosis, deprivation and HoNOS subscales were imputed using multiple imputation by chained equation. The Stata command “mi” was used to create 32 datasets using stimulated values constructed from both covariate and outcome values [26].

## Results

The cohort consisted of 15 210 patients who received a dementia diagnosis in the observation period. Around the dementia diagnosis date, 12 506 (82.2%) were receiving at least one medication with dizziness as a potential side effect: 2588 (20.7%) were on one dizziness-associated medication, 2045 (16.4%) were on two, 3576 (28.6%) were on three to four, 2389 (19.1%) were on five to six and 1908 (15.3%) were on seven or more. Around the dementia diagnosis date, 10 832 (71.2%) patients were receiving at least one medication with hypotension as a potential side effect. Of those, 3020 (27.9%) were on one hypotension-associated medication, 2497 (23.1%) were on two, 3340 (30.8%) were on three to four, 1284 (11.9%) were on five to six and 691 (6.4%) were on seven or more.

Baseline characteristics and comparisons by receipt of dizziness- or hypotension-associated medications are described in Table 1. Patients receiving dizziness-causing medications were significantly younger, from a more deprived neighbourhood and more likely to be from an ethnic minority background and married compared to those not receiving such medications. A higher proportion were recorded with agitated behaviour, depressed mood, psychotic symptoms, moderate-to-severe physical health problems as well as problems with social relationships and occupational/recreational activities. Problematic drug or alcohol use was less common. Those taking medications causing hypotension were less likely to be female and were more likely to be from an ethnic minority background, from a more deprived neighbourhood and with a lower MMSE score than patients not on these medications. They were more likely to have neuropsychiatric symptoms or functional impairment. They more frequently had physical health problems, including recent admissions to general hospitals with cardiovascular disease. Receipt of acetylcholinesterase inhibitors and anticholinergic medications was also more common both in those prescribed medications causing dizziness and in those prescribed medications causing hypotension.

**Table 1:** Sample characteristics and comparisons according to receipt of any dizziness- or hypotension-associated medication.

Characteristics	Whole cohort (*n* = 15 210)	No medication causing dizziness (*n* = 2704)	Receiving any medication causing dizziness (*n* = 12 506)	*P* value^a^ (dizziness)	No medication causing hypotension (*n* = 4378)	Receiving any medication causing hypotension (*n* = 10 832)	*P* value* (hypotension)
Sociodemographic status and cognitive function^b^							
Mean age at dementia diagnosis (SD^c^)	80.9 (8.7)	81.4 (9.2)	80.7 (8.6)	.001	80.9 (8.8)	80.8 (8.7)	.548
Female gender (%)	60.4	61.1	60.2	.371	61.7	59.9	.040
Non-White ethnicity (%)	26.3	24.5	26.7	.020	24.3	27.1	.001
Married or cohabiting status (%)	30.9	26.4	31.9	<.001	30.3	31.1	.336
Mean index of deprivation (SD)	27.3 (11.1)	26.7 (11.4)	27.5 (11.0)	.002	26.1 (11.5)	27.8 (10.9)	<.001
Mean MMSE score at diagnosis (SD)	18.5 (6.3)	18.4 (6.3)	18.6 (6.3)	0.241	18.9 (6.2)	18.4 (6.4)	<.001
HoNOS mental health symptoms (%)^b^							
Agitated behaviour	19.7	13.2	20.9	<.001	11.2	22.9	<.001
Nonaccidental self-injury	1.6	1.1	1.6	.078	0.8	1.9	<.001
Problem-drinking or drug taking	3.4	4.4	3.3	.007	3.7	3.3	.283
Depressed mood	15.5	7.4	17.0	<.001	6.7	18.7	<.001
Hallucinations or delusions	13.9	7.9	15.1	<.001	7.1	16.4	<.001
HoNOS physical illness or disability scale (Comorbidity) (%)^b^				.036			<.001
No or minor problem	43.4	45.8	42.9		54.1	39.5	
Mild problem	27.7	27.2	27.8		25.2	28.6	
Moderate to severe problem	28.9	27.0	29.3		20.8	31.9	
HoNOS functional problems (%)^b^							
Activities of daily living	60.8	62.2	60.6	.162	54.3	63.2	<.001
Living conditions	13.0	14.1	12.8	.119	11.7	13.5	.008
Occupational and recreational activities	32.9	29.8	33.5	.001	26.5	35.2	<.001
Social relationships	16.8	14.4	17.2	.001	11.9	18.6	<.001
Anticholinergic burden according to the Anticholinergic Effect on Cognition scale				<.001			<.001
No central anticholinergic burden	60.9	99.4	52.6		97.8	46.0	
Caution required	21.8	0.5	26.3		1.3	30.0	
Review needed	17.3	0.1	21.1		0.9	24.0	
Any AChEI^d^ prescribed	30.0	3.9	35.6	<.001	28.5	30.6	.011
Hospitalisation with circulatory disease in the past 2 years	27.2	27.5	27.1	0.669	22.3	29.1	<.001

^a^Using a *t*-test/chi-squared test.

^b^Closest to index date.

^c^SD = standard deviation.

^d^AChEI = acetylcholinesterase inhibitor prescribed within 6 months of the index date.

Cox regression analyses for risk of emergency hospitalisation, hospitalised fall and mortality are presented in Table 2. In the follow-up period, 10 737 (70.6%) had at least one emergency hospitalisation, with a median time to first hospitalisation of 1.4 years [interquartile range (IQR) 0.4–4.3]. Considering medications causing dizziness as a continuous variable, each increment in medication number was associated with a 4% increased risk of emergency hospitalisation with little impact of adjustment for covariates. Considering the same exposure as a categorical variable against a nonuser reference group, associations were found in the fully adjusted model from two medications upward. Findings were similar for hypotension-causing medications as an exposure, both as an ordinal and as a categorical exposure.

**Table 2:** The effects of medications associated with dizziness or hypotension on emergency hospitalisation, hospitalised falls and mortality (in Cox regression models).

	Emergency hospitalisation (HR)			Hospitalised fall (HR)			Mortality (HR^c^)		
	Unadjusted	Model 1^a^	Model 2^b^	Unadjusted	Model 1	Model 2	Unadjusted	Model 1	Model 2
Dizziness									
No. of medications causing dizziness as continuous variable	1.04 (1.03, 1.04)^d^	1.04 (1.04, 1.05)	1.04 (1.03, 1.04)	1.00 (0.99, 1.01)	1.01 (0.99, 1.02)	1.00 (0.98, 1.01)	1.01 (1.01, 1.02)	1.02 (1.01, 1.02)	1.00 (0.99, 1.01)
Grouped by number of medications causing dizziness compared to no medication causing dizziness (*n* = 2704)									
One medication causing dizziness (*n* = 2588)	0.92 (0.86, 0.98)	0.94 (0.88, 1.00)	1.02 (0.96, 1.09)	0.89 (0.79, 1.01)	0.87 (0.77, 0.98)	0.91 (0.81, 1.03)	0.82 (0.76, 0.88)	0.81 (0.75, 0.87)	0.92 (0.86, 1.00)
Two medications causing dizziness (*n* = 2045)	0.98 (0.91,1.04)	0.98 (0.92, 1.05)	1.08 (1.00, 1.16)	0.90 (0.79, 1.02)	0.86 (0.75, 0.98)	0.91 (0.80, 1.05)	0.85 (0.79, 0.92)	0.81 (0.74, 0.87)	0.90 (0.83, 0.98)
Three or four medications causing dizziness (*n* = 3576))	1.06 (1.00, 1.12)	1.07 (1.00, 1.14)	1.14 (1.06, 1.21)	0.90 (0.80, 1.00)	0.86 (0.76, 0.96)	0.88 (0.78, 1.00)	0.89 (0.83, 0.95)	0.84 (0.79, 0.91)	0.89 (0.83, 0.96)
Five or six medications causing dizziness (*n* = 2389)	1.13 (1.05, 1.20)	1.13 (1.06, 1.22)	1.17 (1.09, 1.26)	0.90 (0.80, 1.03)	0.88 (0.77, 1.01)	0.89 (0.77, 1.03)	0.96 (0.89, 1.04)	0.91 (0.84, 0.99)	0.93 (0.85, 1.01)
Seven plus medications causing dizziness (*n* = 1908))	1.34 (1.25, 1.43)	1.40 (1.29, 1.51)	1.38 (1.27, 1.50)	0.94 (0.82, 1.07)	0.97 (0.82, 1.13)	0.91 (0.77, 1.07)	1.06 (0.98, 1.14)	1.04 (0.95, 1.14)	0.98 (0.89, 1.08)
Hypotension									
No. of medications causing hypotension as continuous variable	1.05 (1.04, 1.06)	1.06 (1.05, 1.07)	1.04 (1.03, 1.05)	1.01 (0.99, 1.02)	1.02 (1.00, 1.04)	1.00 (0.98, 1.02)	1.03 (1.02, 1.04)	1.04 (1.02, 1.05)	1.01 (0.99, 1.02)
Grouped by number of medications causing hypotension compared to no medication causing hypotension (*n* = 4378)									
One medication causing hypotension (*n* = 2420)	1.13 (1.07, 1.19)	1.12 (1.06, 1.84)	1.13 (1.07, 1.20)	1.06 (0.96, 1.17)	1.00 (0.90, 1.12)	1.00 (0.90, 1.11)	1.09 (1.02, 1.16)	1.03 (0.97, 1.10)	1.02 (0.96, 1.09)
Two medications causing hypotension (*n* = 2051)	1.13 (1.06, 1.20)	1.13 (1.06, 1.20)	1.12 (1.05, 1.19)	1.00 (0.90, 1.12)	0.96 (0.85, 1.08)	0.95 (0.84, 1.07)	1.06 (0.99, 1.14)	0.99 (0.92, 1.07)	0.96 (0.89, 1.03)
Three or four medications causing hypotension (*n* = 2748)	1.21 (1.15, 1.28)	1.21 (1.14, 1.29)	1.16 (1.10, 1.24)	1.00 (0.90, 1.11)	0.96 (0.86, 1.09)	0.92 (0.82, 1.04)	1.15 (1.08, 1.22)	1.08 (1.00, 1.16)	0.98 (0.91, 1.05)
Five or six medications causing hypotension (*n* = 1048)	1.27 (1.17, 1.36)	1.29 (1.19, 1.40)	1.22 (1.12, 1.32)	1.01 (0.87, 1.17)	1.00 (0.85, 1.18)	0.92 (0.78, 1.09)	1.32 (1.21, 1.43)	1.30 (1.19, 1.43)	1.11 (1.01, 1.22)
Seven plus medications causing hypotension (*n* = 608)	1.64 (1.49, 1.80)	1.78 (1.60, 2.00)	1.58 (1.41, 1.76)	1.13 (0.94, 1.35)	1.24 (1.01, 1.54)	1.06 (0.86, 1.32)	1.28 (1.15, 1.43)	1.03 (1.15, 1.48)	1.04 (0.91, 1.19)

^a^Model 1: adjusted for age, gender, ethnicity, marital status, MMSE score, deprivation score and central anticholinergic burden.

^b^Model 2: model 1 plus HoNOS symptom and functioning sores, AChEI prescribed and hospital admission due to serious heart disease in the 2 years before.

^c^HR = hazard ratio.

^d^Bold = *P* < .05.

In total, 2917 (19.2%) patients had at least one hospitalised fall in the follow-up period. However, no relationship was found between this outcome and the number of medications causing dizziness or the number of medications causing hypotension and a hospitalised fall in the fully adjusted model. Likewise, no associations were found with mortality risk for either medication exposure as a continuous variable, and categorical exposures showed no evidence of dose–response associations. The output from generalised estimating equation models of cognitive decline is displayed in Table 3 and also showed no significant associations for either medication exposure with trajectory of cognitive decline.

**Table 3:** Annual cognitive decline and slope differences comparing patients.

	Cognitive decline [MMSE points/year (95% CI)]	Cognitive decline [slope difference (95% CI)]		
		Unadjusted	Model 1^a^	Model 2^b^
Not on any medication causing dizziness (*n* = 1811)	−1.69 (−2.43 to −0.93)	Reference group		
One or more medications causing dizziness (*n* = 10 297)	−1.04 (−1.30 to −0.78)	0.64 (−0.15 to 1.43)	0.73 (−0.06 to 1.52)	0.74 (−0.06 to 1.54)
One medication causing dizziness (*n* = 2047)	−0.19 (−0.77 to 0.39)	1.49 (0.55–2.43)^c^	1.54 (0.60–2.47)	1.49 (0.55–2.43)
Two medications causing dizziness (*n* = 1657)	−0.90 (−1.58 to −0.22)	0.78 (−0.23 to 1.79)	0.82 (−0.19 to 1.83)	0.83 (−0.18 to 1.84)
Three or four medications causing dizziness (*n* = 2941)	−1.56 (−2.03 to −1.09)	0.13 (−0.75 to 1.01)	0.23 (−0.65 to 1.11)	0.26 (−0.63 to 1.15)
Five or six medications causing dizziness (*n* = 2011)	−1.31 (−1.86 to −0.76)	0.37 (−0.56 to 1.30)	0.57 (−0.35 to 1.50)	0.63 (−0.30 to 1.55)
seven plus medications causing dizziness (*n* = 1641)	−1.15 (−1.84 to −0.47)	0.53 (−0.48 to 1.54)	0.62 (−0.40 to 1.63)	0.65 (−0.36 to 1.66)
Not on any medication causing hypotension (*n* = 3233)	−0.75 (−1.22 to −0.27)	Reference group		
One or more medications causing hypotension (*n* = 8875)	−1.25 (1.54 to −0.97)	−0.51 (−1.06 to 0.05)	−0.45 (−0.99 to 0.10)	−0.41 (−0.95 to 0.13)
One medication causing hypotension (*n* = 2420)	−1.24 (−1.62 to −0.53)	−0.49 (−1.10 to 0.11)	−0.29 (−1.00 to 0.42)	−0.29 (−0.99 to 0.42)
Two medications causing hypotension (*n* = 2051)	−1.23 (−1.82 to −0.63)	−0.48 (−1.24 to 0.28)	−0.48 (−1.24 to 0.27)	−0.41 (−1.15 to 0.32)
Three or four medications causing hypotension (*n* = 2748)	−1.47 (−1.94 to −1.00)	−0.73 (−1.40 to −0.06)	−0.57 (−1.23 to 0.10)	−0.49 (−1.14 to 0.17)
Five or six medications causing hypotension (*n* = 1048)	−1.27 (−2.22 to −0.32)	−0.53 (−1.59 to 0.54)	−0.47 (−1.53 to 0.60)	−0.49 (−1.52 to 0.55)
Seven plus medications causing hypotension (*n* = 608)	−1.24 (−2.43 to −0.13)	−0.49 (−1.70 to 0.72)	−0.55 (−1.76 to 0.65)	−0.38 (−1.57 to 0.80)

^a^Model 1: adjusted for age, gender, ethnicity, marital status, deprivation score and central anticholinergic burden.

^b^Model 2: model 1 plus HoNOS symptom and functioning sores, AChEI prescribed, and hospital admission due to serious heart disease in the 2 years before.

^c^Bold = *P* < .05 for slope difference in the respective models.

Given the associations of both exposures with hospitalisations but not with hospitalised falls, supplementary analyses were carried out to further investigate the associations between medications and specific causes of hospitalisation. Considering the seven most common primary discharge diagnoses grouped by ICD-10 chapter code (see Table 4), in fully adjusted analyses, a significant increased risk for all hospitalisation causes was associated with incremental numbers of medications in either exposure category, apart from an absent association between hypotension-causing drugs and musculoskeletal hospitalisations. The strongest associations were with circulatory disease as the primary cause of hospitalisation.

**Table 4:** Other causes of hospitalisation and their relationship with the number of medications prescribed in adjusted Cox regression models.

Cause of hospitalisation (by ICD-10 chapter)	Proportion of patients with cause-specific hospitalisations in whole cohort (*n* = 15,210)	Hazard ratio per unit increase in number of medications causing dizziness (95% confidence interval)			Hazard ratio per unit increase in number of medications causing hypotension (95% confidence interval)		
		Unadjusted	Model 1^a^	Model 2^b^	Unadjusted	Model 1	Model 2
R (symptoms, signs and abnormal clinical and laboratory findings, not elsewhere classified)	27.5% (*n* = 4186)	1.04 (1.03, 1.05)^c^	1.05 (1.04, 1.06)	1.03 (1.02, 1.05)	1.06 (1.04, 1.07)	1.07 (1.05, 1.08)	1.04 (1.02, 1.06)
J (diseases of the respiratory system)	25.1% (*n* = 3824)	1.05 (1.04, 1.06)	1.05 (1.04, 1.07)	1.04 (1.02, 1.05)	1.07 (1.06, 1.08)	1.07 (1.05, 1.09)	1.04 (1.02, 1.06)
N (diseases of the genitourinary system)	23.3% (*n* = 3534)	1.04 (1.03, 1.05)	1.04 (1.03, 1.06)	1.03 (1.02, 1.04)	1.06 (1.04, 1.07)	1.06 (1.05, 1.08)	1.04 (1.01, 1.05)
S (injuries)	21.1% (*n* = 3212)	1.01 (1.00, 1.03)	1.03 (1.01, 1.04)	1.02 (1.00, 1.03)	1.02 (1.01, 1.04)	1.04 (1.02, 1.06)	1.02 (1.00, 1.04)
I (diseases of the circulatory system)	18.8% (*n* = 2856)	1.05 (1.04, 1.06)	1.07 (1.06, 1.09)	1.06 (1.04, 1.07)	1.07 (1.05, 1.08)	1.10 (1.08, 1.12)	1.07 (1.05, 1.09)
K (diseases of the digestive system)	15.5% (*n* = 2351)	1.04 (1.03, 1.06)	1.04 (1.02, 1.06)	1.03 (1.02, 1.05)	1.04 (1.02, 1.06)	1.03 (1.01, 1.06)	1.02 (1.00, 1.05)
M (diseases of the musculoskeletal system and connective tissue)	9.8% (*n* = 1495)	1.02 (1.01, 1.04)	1.03 (1.01, 1.05)	1.02 (1.00, 1.04)	1.02 (1.00, 1.05)	1.03 (1.01, 1.06)	1.01 (1.00, 1.04)

^a^Model 1: adjusted for age, gender, ethnicity, marital status, MMSE score, deprivation score and central anticholinergic burden.

^b^Model 2: model 1 plus HoNOS symptom and functioning sores, AChEI prescribed and hospital admission due to serious heart disease in the 2 years before.

^c^Bold = *P* < .05.

## Discussion

The present study investigated the use of medications with dizziness or hypotension as a side effect in a cohort of 15 210 older adults with a diagnosis of dementia and the extent to which this was associated with subsequent adverse outcomes of hospitalisation, hospitalised falls, mortality and cognitive decline.

Cumulative numbers of medications received with dizziness or hypotension as a side effect were associated with an incremental 4% increased risk of emergency hospitalisation. At the upper end of the exposure distribution, patients receiving seven or more medications causing dizziness were found to have a 38% increased risk of emergency hospitalisation (compared to those receiving none) and those with seven or more medications causing hypotension had a 58% increased risk of emergency hospitalisation. While these findings align with previous research that has linked dizziness to hospitalisation [27] and polypharmacy to a higher risk of hospitalisation [28], we believe that this study is the first to report a direct association between polypharmacy specifically of dizziness-associated medications and an increased risk of emergency hospitalisation.

Within older adult populations, polypharmacy has been found to be associated with falls [29] and dizziness has been identified to be an independent predictor of future falls [9]. Unexpectedly, in our study no associations were found between medication exposure and hospitalised falls. However, another retrospective investigation of emergency department admission in older adults identified 64 different discharge diagnoses linked to the symptom of dizziness [27]. Therefore, it is reasonable to anticipate that dizziness may influence the risk of hospitalisation for a variety of reason. In support of this, our supplementary analyses indicated nonspecific associations between a unit increase of medication causing dizziness or hypotension and seven different causes of hospitalisation, although circulatory diseases were the most common.

As a symptom, dizziness is complicated, and medications causing dizziness may be one of multiple factors contributing to an outcome of falls. Multiple intrinsic and extrinsic risk factors for falls in people with dementia have been identified [30, 31]. Associations may vary by community or institutional residence [30] and dementia is itself associated with increased risk of falls [32], particularly vascular dementia, mixed dementia and dementia in other diseases in comparison to Alzheimer’s disease [31]. Specific associations of medication use with hospitalised falls might be obscured by both the range of other factors contributing to that outcome and the range of outcomes associated with dizziness. Furthermore, although the current study adjusted for a range of covariates, residual confounding cannot be ruled out entirely and adverse outcomes might be potentially obscured by differential prescribing (medications less likely to be initiated in people judged to be at risk).

No associations were evident with mortality as an outcome. Although polypharmacy has been found to be associated with increased risk of mortality [33], a Swedish cohort study found that some medications were only weakly associated with mortality risk [34]; therefore, it is possible that associations were obscured because of the profile of the medications scaled. Another potential reason for our findings is that mortality risk is more strongly related to underlying health conditions [34], and that unmeasured confounding obscured the association of interest. Likewise, for cognitive decline, although a previous study identified an association between dizziness and attentional and visuospatial cognitive abilities [35], there was no clear association observed for this outcome, which might reflect obscuring competing causes and/or selective, more cautious prescribing. Furthermore, cognitive decline in this study was assessed using MMSE total scores and specific subdomains of cognitive function were not investigated. Further research should look to investigate whether medications with dizziness and/or hypotension as a side effect are associated with subdomains of cognitive functions.

The present study tested its hypotheses using data from a large, naturalistic sample representing a higher proportion of cases of dementia than would be possible in a traditional recruited cohort. Considering the sample size, a relatively high level of granularity was afforded, allowing access to a range of confounding factors such as physical illness, prior hospitalisations and medication use, as well as the medication exposures. Linkage to national datasets for mortality and hospitalisation outcomes enabled near-complete follow-up.

Despite the aforementioned strengths, the limitations of using such data need to be considered. Although a range of covariates were considered and adjusted for, there may be residual confounders that were not captured; these include the severity of comorbidities and the physician’s prescribing behaviour, as well as use of over-the-counter medications. Secondly, dizziness is a common side effect of multiple medications, and the complications of polypharmacy in the older population can make it difficult to identify whether medications themselves are causing the dizziness or whether it is a product of their interactions, which we did not attempt to quantify. The limitations of the data source also need to be considered. Information on coprescribed medications was assembled following referral to memory assessment services. Therefore, medications prescribed prior to the referral and subsequent changes were not captured. Additionally, information on the dosage and duration of medication use was not assessed within the analysis. Fall-related hospitalisation was identified through structured discharge diagnoses and was thus limited to severe falls which led to hospitalisation and required treatment. Less severe and nonhospitalised falls were not considered; therefore, the full scope of falls as an outcome may not have been captured within this study. Finally, comorbid physical health status was measured using the HoNOS subscale, which does not provide details on specific long-term [12] or comorbid conditions.

## Conclusion

Within individuals with dementia, polypharmacy [3] and ADRs [5] are frequent phenomena. Dizziness is a common medication side effect [7] and has itself been linked to hospitalisations and falls [9, 27]. Findings from this cohort study suggest that the number of medications received with a side effect of dizziness or hypotension is associated with an incrementally increased risk of emergency hospitalisation across a range of common discharge diagnoses and not specifically fall-related hospitalisations. No consistent associations were found with mortality or subsequent cognitive decline. The findings highlight the complexity of dizziness as a side effect. Enhancing knowledge on the outcomes of medications with dizziness as a side effect may assist in minimising risk events particularly in people with dementia and patients who are more likely to receive polypharmacy. These findings provide empirical evidence and can be integrated into clinical decision-making tools such as Medichec to help identify individuals who might be at higher risks of ADRs. As mentioned in Methods, Medichec is an application that provides information on the adverse effects of medication [15]. This can assist in reducing challenges of medication management in people with dementia. Further research is needed to detangle other medication properties, coprescribing profiles and adverse outcomes.

## Data Availability

All the relevant aggregate data are found within the article. The data used in this work have been obtained from CRIS, a system that has been developed for use within the NIHR Mental Health Biomedical Research Centre at SLaM. It provides authorised researchers regulated access to anonymised information extracted from SLaM's electronic clinical records system. Individual-level data are restricted in accordance with the strict patient-led governance established at SLaM, and by NHS Digital for the case of linked data. Data are available for researchers who meet the criteria for access to this restricted data: (i) SLaM employees or (ii) those having an honorary contract or letter of access from the trust. For further details, and to obtain an honorary research contract or a letter of access, contact the CRIS administrator at cris.administrator@kcl.ac.uk.
